# Audiologists should not fail with falls: A call to commit to prevention of falls in older adults

**DOI:** 10.4102/sajcd.v68i1.841

**Published:** 2021-09-30

**Authors:** Christine Rogers

**Affiliations:** 1 Department of Health and Rehabilitation Sciences, Division of Communication Sciences and Disorders, Faculty of Health Sciences, University of Cape Town, Cape Town, South Africa

**Keywords:** falls, fall prevention, fall risk, older adults, hearing impairment, audiology

## Abstract

Globally, falls are a serious economic and public health concern. While all age groups are impacted by falls, the threats to morbidity and mortality are most severe in older adults. Recent literature has linked hearing loss, and related issues such as an increase in sedentary behaviour, to a greater risk of falls. Therefore, this opinion article aims to raise audiologists’ awareness of falls in ageing patients or clients, and calls for change in terms of having these rehabilitation professionals embrace identification and management of fall risk.

Population ageing is arguably one of the most significant social transformations of the 21st century with direct and obvious implications to healthcare and health policy:

… Ageing, of course, is inevitable and older people are more likely to have multiple, coexistent, and interrelated health problems. This fact, together with geriatric syndromes, frailty and impaired cognition, continence, *gait, and balance*, suggests the need for a more thorough ‘retooling’ of the healthcare system and workforce to meet the health challenge of ageing. (Stucki, Bickenbach, Gutenbrunner, & Melvin, 2018, p. 309) (Italicised by author)

Globally, falls are a serious economic and public health concern (Haddad, Bergen, & Florence, 2019). Injurious falls present a significant morbidity and mortality threat to the lifespan from early infancy (Alonge & Hyder, 2014) to young, middle (Verma et al., 2016) and late adulthood (Shen et al., 2017). A fall describes an individual unintentionally and involuntarily moving towards the ground or other lower level (Lima, Ricci, Nogueira, & Perracini, 2018), which is not the result of a major intrinsic event or hazard (Lamb, Jørstad-Stein, Hauer, & Becker, 2005). Intrinsic events are idiosyncratic health-related issues, such as visual acuity or balance deficits (Gazibara et al., 2017), which may include age, sex and ethnicity (Kosma, 2014). This opinion article aims to raise audiologists’ awareness of falls in ageing patients or clients and calls for change in terms of identifying and addressing the risk of falls.

Falls are an important clinical concern for older adults, associated with increased morbidity and mortality rates, as well as having major implications for public health (Gazibara et al., 2017). As the world’s population ages, the rate of falls is growing disproportionally. The ranking of fall-related deaths has increased over some decades, with the World Health Organisation (WHO) confirming that falls are the second leading cause of deaths and unintentional injury deaths after road traffic accidents worldwide (WHO, 2018). In the United States of America, falls are the predominant cause of injury across almost all ages (Timsina et al., 2017). An older adult in America falls every second, requires emergency services for fall-related injuries every 18 s with a fall-linked death being reported every 20 minutes (CDC, 2016). Low- and-middle-income countries are neither spared the phenomenon of injurious falls nor ageing. Data from 17 countries across four different income rankings suggest that falls are associated with rural settings, increasing age,^1^ female sex and current or previous alcohol use (Raina et al., 2016). In South Africa, alcohol represents a significant health and social problem, with one of every three adults consuming alcohol and one in seven binge drinking (Vellios & Van Walbeek, 2018). Intrinsic fall risk factors already described, some of which are linked to social determinants of health, combine with environmental hazards, such as poor infrastructure in public places, and should result in placing falls firmly on the agendas of rehabilitation professionals.

## Relevance of falls to audiologists

### Hearing loss is linked to falls

As humans age, sensorineural hearing loss (presbycusis) develops (Nordvik et al., 2018). Meta-analysis has suggested that hearing loss established with pure tone audiometry results in an almost seven-fold increased risk of falling (Jiam, Li, & Agrawal, 2016). Other correlates of hearing impairment in older adults relevant to an increased fall risk include sedentary behaviour, slower gait speed (Shayman, Earhart, & Hullar, 2017), social isolation and withdrawal (Criter & Honaker, 2013), and cognitive decline (Rutherford, Brewster, Golub, Kim, & Roose, 2017). With an ageing population, the number and rate of individuals seeking audiology services will increase (Criter & Honaker, 2016). Thus, audiologists have extensive access to older adults, and are in a pivotal position to screen for falls and associated risk factors. Despite the opportunity for audiologists to act as gatekeepers for identifying the risk of falls in their hearing-impaired patients, disappointingly in practice, at least in the United States of America, less than one-third attempt any form of screening (Criter & Honaker, 2017).

### Holistic balance assessment

Historically, audiologists had a role in assessing vestibular integrity, likely because of the location of the end organs in the inner ear and correlations between labyrinthine disorders, which may affect both hearing and balance. Internationally, increasing numbers of audiologists are involved in the assessment and management of patients with vestibular dysfunction (Nelson, Akin, Riska, Andresen, & Mondelli, 2016). In South Africa, services include audiological assessments, clinical and instrumented vestibular evaluations, and vestibular rehabilitation therapy (Seedat, Khoza-Shangase, & Sebothoma, 2018).

Professional scope of audiology mandates that virtually all physical outcome measures related to fall risk assessment would be included. More advanced testing, for example, using the MiniBESTest to explore the different constructs of balance and fall risk (Magnani et al., 2020), would require more in-depth training and some experience. In 2011, South African audiology degree training programmes were directed to include vestibular management as an exit level outcome (HPCSA, 2018). Notwithstanding the necessity for graduate professionals to have skills in vestibular management, audiologists in South Africa have been slow to embrace vestibular practice (Seedat et al., 2018). It is possible that by starting with a small but patient-valued service, such as screening older adults for fall risk, professional confidence could be built, and audiologists would be inspired to engage more fully in this critical area.

### Dizziness and vertigo increase fall risk

Dizziness and vertigo are common complaints reported in older populations (Hall & Meldrum, 2016), some of whom consult an audiologist, who may function either in a team of professionals or as the sole provider. Numerous researchers have described the link between vestibular deficits and falls. For example, a case-control study reported that 80% of older adults with clinically significant vestibular impairment fell (Liston et al., 2014). Even if participants’ symptoms of dizziness had settled because of compensatory processes, their fall risk remained elevated (Liston et al., 2014). Another study of patients with vestibular impairment and associated dizziness suggested an increased risk of falls by a factor of 12.3 (Allen, Ribeiro, Arshad, & Seemungal, 2017). Overall, these and other studies support the view that audiologists should be questioning all older adults regarding falls, not just those who complain of dizziness. Indeed, it is argued that because of the predictability of falls, failing to evaluate older adults’ risk of fall is a significant neglect in the duty of care to audiology patients.

### Calls for agency and change

The key roles of an audiologist include disease prevention and health promotion activities at all levels of healthcare. Decades ago, calls were made for audiologists to concern themselves with the ‘balance’ system rather than purely vestibular function and evaluation (Jacobson, 2001). Leaders such as Jacobson and other audiologists continue to remind us to embrace balance and fall issues; however seemingly, little progress has been made in changing the clinical practice of audiologists.

A directive for audiologists performing vestibular assessments with American Medicare beneficiaries to conduct fall risk assessment and formulate plans of care (Bassett & Honaker, 2016) offers a glimmer of hope. However, much work remains to encourage more widespread adoption of plans for fall prevention and health promotion activities within our profession. Indeed, the focus in emerging regions needs to shift from curative services to health promotion at all ages and levels of care.

### Moving towards rehabilitation

The profession experiences a challenge because audiologists do not necessarily embrace rehabilitation (Hull, 2018) as do other professionals, such as physio- and occupational therapists (Babatunde, MacDermid, & MacIntyre, 2017). Current practice has a diagnostic focus, rather than deeply engaging with patients and building strong therapeutic alliances (Hull, 2018). There is convincing evidence that vestibular rehabilitation is effective in managing symptoms of vestibular deficits (Dunlap, Holmberg, & Whitney, 2019). Thus, programmes based on vestibular rehabilitation and applied in a fall prevention context could be argued to be relevant to audiologists and their patients. Moreover, many effective exercise-based fall prevention programmes exist, with compelling evidence to support them. Here, audiologists can play a role by discussing the impact of hearing loss and its associated sedentary behaviour and withdrawal, raising awareness in their patient populations and encouraging physical activity (PA) and appropriate referral to community resources. More detailed strategies are discussed in the following section.

## Steps audiologists should implement to identify and manage fall risk

The second part of this opinion article suggests simple strategies for audiologists to identify and manage fall risk amongst older adult patients or clients.

### Prescribe activity

Interestingly, a large number of research studies on fall risk focus on factors that are not responsive to therapeutic interventions, for example, chronological age and sex. Otherwise, attention is directed to medical management like medication review. Issues such as activity-based strategies for fall risk management have received less attention. For example, few authors have examined sedentary behaviour (as previously observed, associated with both ageing and hearing loss) and found that challenging this lifestyle with PA is a potentially treatable cause of falls (Thibaud et al., 2012). Thibaud et al. (2012) considered the role of PA and falls by meta-analysis. The results revealed that for older adults, PA is a protective factor for falls, particularly injurious falls. Physical activity may address intrinsic risk factors for falls by improving balance and gait speed, muscular and physical strength. Therefore, PA should be recommended in the management of fall risk. Furthermore, Cochrane reviews have addressed multiple interventions to fall risk, including exercise-based fall prevention programmes, with evidence in favour of the latter (Gillespie et al., 2012; Sherrington et al., 2019). A systematic review by Sherrington et al. (2020) was undertaken to inform the WHO guidelines on PA and sedentary behaviour, which re-affirmed the value of exercise, particularly balance-related activities including Tai Chi, to reduce the risk of falls in older adults (Sherrington et al., 2020). If audiologists are empowered with quality information, they are better informed to educate their older adult populations.

### Screen for fall risk

Numerous instruments exist to screen for falls and fall risk. Ideally, previous falls, fear of falling, and the number of prescribed medications should be ascertained and noted along with the age and sex of the patient. Polypharmacy is the simultaneous use of multiple drugs by an individual and is present in approximately half of all older adults from high-income countries (Morin, Johnell, Laroche, Fastbom, & Wastesson, 2018). There is little reason to believe that the narrative is any different in South Africa (Saka, Oosthuizen, & Nlooto, 2019). Thresholds for the number of medications that constitute polypharmacy vary but are usually between three and six (Gutiérrez-Valencia et al., 2018). There are increasing links between polypharmacy, falls and the onset of frailty (Gutiérrez-Valencia et al., 2018; Wastesson, Morin, Tan, & Johnell, 2018). Fear of falling may occur despite no recent fall and is a powerful risk factor for future falls, likely because of self-restriction and cautious gait (Auais et al., 2017; Denkinger, Lukas, Nikolaus, & Hauer, 2015; Lavedán et al., 2018). Fear of falling may in fact be enabling if an older adult is willing to explore strategies to address the fear, such as embarking on an exercise programme. A fall reported within the previous year similarly increases the risk of another fall, and thus, helpful information is obtained from simple questioning (Lusardi et al., 2017).

### Physical outcome measures for falls

There are four tests suitable for everyday clinical practice that have evidence to support them (Lusardi et al., 2017). These include the Timed Up and Go (TUG) test, which will be discussed here, the Five Times Sit to Stand Test, Single Leg Stance Test and preferred walking speed; however, more research studies are required to definitively link them to fall risk. Briefly, the TUG test requires an individual to get up from a chair, walk 3 m around a traffic cone, return and sit down again. Several balance-related activities are required, such as transitioning from sitting to standing, changing direction around an obstacle and ambulation. The test is timed, and age- and sex-matched normative data are available (Bohannon, 2006; Ibrahim, Singh, & Shahar, 2017; Pondal & Del Ser, 2008). The TUG is recommended for primary care by the American and British Geriatric Societies (Smith et al., 2016), and can be administered quickly and efficiently. Variations for the TUG exist (manual and cognitive) and have promise for refining fall risk. Individuals whose result (time to complete TUG in seconds) falls outside the Centers for Disease Control and Prevention’s (CDC) 12 s cut-off should be considered at risk of falls and referred appropriately. A copy of the test procedure can be downloaded from https://www.cdc.gov/steadi/pdf/TUG_test-print.pdf.

### Manage holistically

Consider referral back to the primary care provider with the results of the audiological assessment, which might suggest an increased risk of falls. Steps such as medication review may be advisable. Clinical assessment of vestibular function, falls history and risk, and balance and gait (either with an audiologist with a special interest or a physiotherapist) could be indicated. Resources such as the CDC’s Stopping Elderly Accidents, Deaths and Injuries (STEADI) programme are helpful (https://www.cdc.gov/steadi/index.html) and provide information for patients and healthcare professionals. Checklists to manage potential fall hazards at home are appreciated by patients and their families alike and are freely available on the internet. Call systems in case of falls are useful for older adults who live alone. Finally, a pamphlet demonstrating how to get up after a fall (e.g. see https://www.stayonyourfeet.com.au/wp-content/uploads/2016/01/SOYF-Up-Off-the-Floor-knees-v1-lowres.pdf) or a video link can be empowering if patients practice how to get up safely after a fall.

## Conclusion

Audiologists have multiple roles, including being rehabilitation professionals, enhancing disease prevention and health promotion activities, and being advocates of change. First, compelling evidence exists to support that exercise is ‘medicine’ (Sallis, 2009), with the implication that all healthcare professionals should be prescribing it. Second, the case for exercise and PA to reduce the risk of falls is strong. Specific to fall risk, which is enhanced by factors such as ageing and hearing loss, audiologists are ideally situated as gatekeepers and should be an additional entry point to management of older adult populations with falls. Screening for falls is simple and quick, even in constrained settings; however, the impact of falls is life-changing for individuals who fall and their families. As Africa greys, let us embrace our role and start identifying and managing the risk of fall amongst the older adult populations!

Until the great mass of the people shall be filled with the sense of responsibility for each other’s welfare, social justice can never be attained. (Keller, 1914)

## Footnotes

1 Note the referenced study only considered ages between 30 and 70 years.
